# Schizophrenia Increases Variability of the Central Antioxidant System: A Meta-Analysis of Variance From MRS Studies of Glutathione

**DOI:** 10.3389/fpsyt.2021.796466

**Published:** 2021-11-30

**Authors:** Lena Palaniyappan, Priyadharshini Sabesan, Xuan Li, Qiang Luo

**Affiliations:** ^1^Department of Psychiatry, University of Western Ontario, London, ON, Canada; ^2^Robarts Research Institute, London, ON, Canada; ^3^Lawson Health Research Institute, London, ON, Canada; ^4^Department of Medical Biophysics, University of Western Ontario, London, ON, Canada; ^5^MOE-Key Laboratory of Computational Neuroscience and Brain-Inspired Intelligence, Institute of Science and Technology for Brain-Inspired Intelligence, Fudan University, Shanghai, China; ^6^National Clinical Research Center for Aging and Medicine at Huashan Hospital, State Key Laboratory of Medical Neurobiology and MOE Frontiers Center for Brain Science, Institutes of Brain Science and Human Phenome Institute, Fudan University, Shanghai, China

**Keywords:** psychosis, redox, anterior cingulate (ACC), n-acetyl cysteine (NAC), Nrf2, spectroscopy

## Abstract

Patients with schizophrenia diverge in their clinical trajectories. Such diverge outcomes may result from the resilience provided by antioxidant response system centered on glutathione (GSH). Proton Magnetic Resonance Spectroscopy (1H-MRS) has enabled the precise *in vivo* measurement of intracortical GSH; but individual studies report highly variable results even when GSH levels are measured from the same brain region. This inconsistency could be due to the presence of distinct subgroups of schizophrenia with varying GSH-levels. At present, we do not know if schizophrenia increases the interindividual variability of intracortical GSH relative to matched healthy individuals. We reviewed all 1H-MRS GSH studies in schizophrenia focused on the Anterior Cingulate Cortex published until August 2021. We estimated the relative variability of ACC GSH levels in patients compared to control groups using the variability ratio (VR) and coefficient of variation ratio (CVR). The presence of schizophrenia significantly increases the variability of intracortical GSH in the ACC (logVR = 0.12; 95% CI: 0.03–0.21; log CVR = 0.15; 95% *CI* = 0.06–0.23). Insofar as increased within-group variability (heterogeneity) could result from the existence of subtypes, our results call for a careful examination of intracortical GSH distribution in schizophrenia to seek redox-deficient and redox-sufficient subgroups. An increase in GSH variability among patients also indicate that the within-group predictability of adaptive response to oxidative stress may be lower in schizophrenia. Uncovering the origins of this illness-related reduction in the redox system stability may provide novel treatment targets in schizophrenia.

## Introduction

Glutathione is an antioxidant molecule seen as a fundamental component in the response to oxidative stress in the brain (1). Animal models that mimic the phenotype of schizophrenia have been shown to have low central glutathione levels (2, 3). These pre-clinical studies have predicted reduced brain glutathione in schizophrenia, which when corrected by using GSH precursors or drugs that enhance the genetic regulation of GSH transcription [*via* Nrf2 pathways (4)] could reduce symptoms of schizophrenia. However, *in vivo* measurement of GSH from the brain using Magnetic Resonance Spectroscopy (MRS) has produced inconsistent results to date. Some studies have indicated the presence of a small to medium sized reduction, while others have noted no differences, or higher GSH in schizophrenia (5, 6). This has raised the speculation that there may be two subgroups of patients with schizophrenia; one with increased and the other with decreased GSH levels (7). To date, there is no empirical evidence to support this hypothesis of intracortical GSH variability in schizophrenia.

In recent times, meta-analysis of group variance has emerged as an important method in the investigation of therapeutic (8, 9) and neurobiological (10, 11) heterogeneity in schizophrenia. This approach looks beyond synthesizing effect sizes (mean differences) from pooled group comparisons. Instead, by comparing group variances we can test if distinct subgroups or more pronounced individual differences occur in patients relative to healthy subjects. To date, meta-analyses have established a small reduction in GSH levels in the anterior cingulate cortex (ACC), the most sampled site for MRS studies on GSH in schizophrenia, by averaging mean GSH values (7, 12). In the present study, we examine the systematic changes in the variability of ACC GSH among patients with schizophrenia.

Greater variability in GSH levels in schizophrenia would mean that some patients (i.e., the putative low-GSH subgroup) are more likely to benefit from the use of GSH precursors or drugs that activate Nrf2 pathway than the others. In other words, the outcomes of antioxidant trials are likely to be more robust when the treatment is stratified for the GSH-based subgroups. Identifying GSH-based subgroups will also enable more precise mechanistic investigations of the antioxidant system in schizophrenia.

## Methods

### Search Process

We followed the search process set out in our previous meta-analysis ACC GSH studies (7) and extended the time line to 31 August 2021 and searched MEDLINE electronic database. We used the following search terms: (schizophrenia OR schizo^*^ OR psychos^*^ OR psychot^*^) AND (“1H-MRS” OR “1H NMRS” OR “1HMRS” OR “MRS” OR “Magnetic resonance spectroscopy” OR “Spectroscopy” OR “proton magnetic resonance spectroscopy”) AND (“glutathione” OR “GSH”). To include studies reporting GSH as a secondary measure, we used the terms (“myoinositol” OR “NAA” OR “n-acetyl aspartate” OR “glutamine” OR “neurometabolic” OR “Glutamate” OR “Glu” OR “GABA” OR “Lactate” OR “creatinine”) and examined the results for reported GSH values. Next, we repeated the search with Google Scholar and EMBASE (through Ovid search engine) to identify journal articles that were not indexed on MEDLINE. We also undertook a manual search of reference lists of review articles and eligible full text articles. Finally, as in our prior study, we also searched the citation records of Google Scholar for all identified full text articles in order to locate in press and pre-print articles that are not yet indexed. Two authors undertook independent searches using the inclusion and exclusion criteria without any exchange of notes. This followed the guidelines set out by the consensus statement from PRISMA group (13). The protocol for this updated review has not been previously published or registered elsewhere. The inclusion and exclusion criteria were the same as used by Das et al. (7). In brief, all peer-reviewed articles in English language reporting GSH concentrations in the brain in patients with schizophrenia or schizoaffective disorder in comparison with a healthy control group were included. As in our prior work, we only included those studies with the largest proportion of MRS voxel placed on the anterior cingulate region of the medial prefrontal cortex, anterior to the posterior commissure, as per the cingulate boundaries defined by Vogt et al. (14). We excluded the following studies: (1) those reporting only within-subject changes in GSH (2) studies that excluded adult samples of age >16. If a single study was reported as two samples, the largest sample was included. We utilized diagnostic group-wise data provided by the study authors when the original studies reported GSH data from non-diagnostic clusters (15).

We identified seven additional eligible datasets, along with the 13 located in 2018 (details below). We used meta-analysis of variance to investigate inter-individual variability in GSH levels in the anterior cingulate cortex in patients with schizophrenia or schizoaffective disorder compared to healthy controls. We included 20 studies that reported measurements of GSH levels in 589 patients and 601 controls and extracted research-specific means and standard deviations of 1HMRS GSH concentrations in the patient groups and control. This search procedure was a continuation of our previous efforts reported in Das et al. (7). Each study was independently extracted by at least 2 raters and recorded in an Excel sheet. A table with the demographic details of the studies before 2018 can be found in Das et al. (7); for studies after 2018, see Table 1 in a recent review discussing challenges and opportunities related to glutathione in schizophrenia (16).

Variation Ratio (VR) and Coefficient of Variation Ratio (CVR) were used to quantify variation differences (17). Our first variability outcome measure is the natural logarithm of variability ratio (logVR), where VR is the ratio of standard deviation estimates between the patients and controls, as shown below:


$$\begin{array}{l} \text{logVR}=\log\left(\frac{{\hat{\sigma }}_{p}}{{\hat{\sigma }}_{c}}\right)=\log\left(\frac{s_{p}}{s_{c}}\right)+\frac{1}{2\left(n_{p}-1\right)}-\frac{1}{2\left(n_{c}-1\right)}, \end{array}$$

where ${\hat{\sigma }}_{p}$ and ${\hat{\sigma }}_{c}$ are estimates of population standard deviations (SDs), *s*_*p*_ and *s*_*c*_ are reported sample SDs, and *n*_*p*_ and *n*_*c*_ are sample sizes of the patient group and the control group, respectively.

The first measure focuses only on the variability of the internal measure values in the two groups, and we also report the results of the analysis using the natural logarithm of the coefficient of variation (logCVR), where the CVR is the ratio of the estimated total coefficient of variation between the patient and the control group, as an outcome measure. This measure quantifies the difference in variability after scaling to the mean of each group. ln CVR can be determined as follows:


$$\begin{array}{l} \text{logCVR}=\log\left(\frac{{\hat{\sigma }}_{p}/\;{\bar{x}}_{p}}{{\hat{\sigma }}_{c}/\;{\bar{x}}_{c}}\right)\\ \;=\log\left(\frac{s_{p}/\;{\bar{x}}_{p}}{s_{c}/\;{\bar{x}}_{c}}\right)+\frac{1}{2\left(n_{p}-1\right)}-\frac{1}{2\left(n_{c}-1\right)}, \end{array}$$

where ${\bar{x}}_{p}$ and ${\bar{x}}_{c}$ are reported sample means for patient and control groups, respectively. A logVR or logCVR of 0 indicates equal variability between patients and controls, >0 indicates greater variability in patients, and <0 indicates lower variability in patients.

We also used the standardized mean difference with heteroscedastic population variance (SMDH) to estimate the effect size of Hedges' g. Hedges' g effect values of 0.20, 0.50 and 0.80 respectively represent small, medium and large effects (18).

Meta-analysis was performed using R Studio's Metafor package (19). We used random-effects model to calculate the pooled effect size, with the 95% confidence limit. This approach can provide more reliable inferences when there is significant heterogeneity between individual studies. We used *I*^2^ statistics for quantification and Cochran's Q for statistical significance test to evaluate heterogeneity.

## Results

### Search Results

A total of 17 studies were identified by literature search [one with two eligible contrasts (20) and one with three eligible contrasts (21), making a total of 20 datasets] in schizophrenia (6, 15, 20–34). All these studies were published between 2001 and 2021, and included a total of 589 patients and 601 controls. The sample sizes ranged from 9 to 88 for controls and 11 to 74 for patients.

### Pooled Effect-Size

Between-studies heterogeneity was not significant for VR (**I**^**2**^ = 12.8%, Cochran's *Q* = 24.14, *p* = 0.19) and CVR (**I**^**2**^ = 0%, Cochran's *Q* = 14.33, *p* = 0.76) estimates, but as expected, for SMDH there was a significant between-studies heterogeneity (**I**^**2**^ = 47.4%, Cochran's *Q* = 37.15, *p* = 0.008).

**Figure 1:**
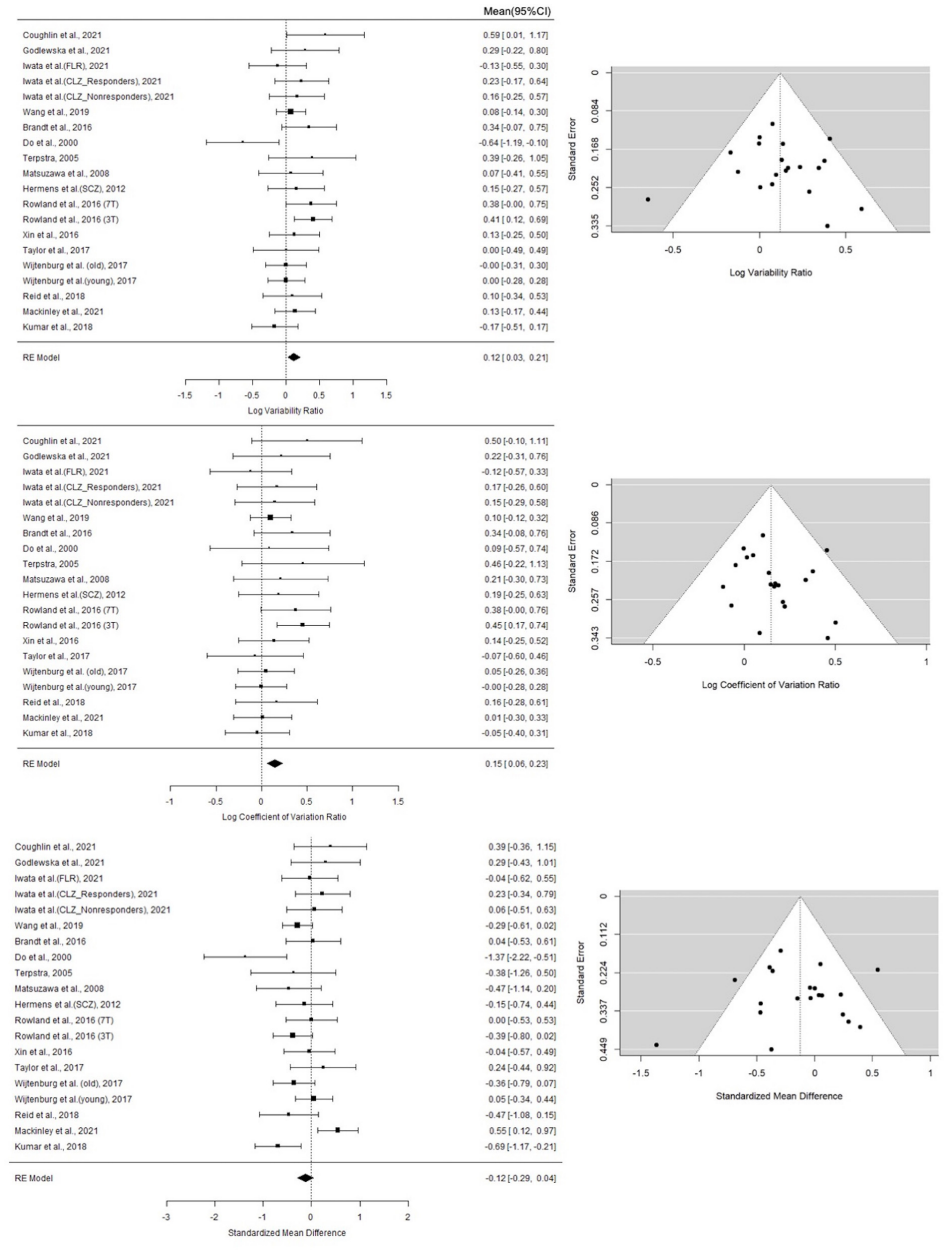
Forest plots of studies for variability ratio (VR) and coefficient of variation ratio (CVR) and standardized mean difference with heteroscedastic population variance (SMDH) of GSH levels in schizophrenia vs. healthy subjects. First column lists the first author name and year of the studies from which the data point for analysis were obtained. For studies that yielded more than one contrast, the characteristic feature separating the contrasts are given in brackets. The last column of the Forest Plots lists the mean and 95% CI of the estimates. The summary statistics based on Random effects model is provided for each synthesis at the bottom of the list. Funnel Plots for each metric are also displayed. Statistical tests for publication bias were not significant; these are reported in the Results section.

A random-effect analysis showed increased variance in GSH levels in patients with schizophrenia compared to healthy controls (logVR = 0.12; 95% CI: 0.03–0.21; log CVR = 0.15; 95% CI = 0.06–0.23). When the SMDH method was used, the mean GSH levels had no significant difference between the two groups (SMDH = −0.12; 95% CI: −0.29–0.04). These results are displayed in the forest plot in Figure 1. On visual examination (Figure 1), the funnel plots for the three metrics were symmetric, and the Begg's test and the Egger's test for publication bias were not statistically significant (logVR: 0.7246 for Begg's test; 0.7993 for Egger's test; log CVR: 0.2884 for Begg's test; 0.4453 for Egger's test; SMDH: 0.6771 for Begg's test; 0.6791 for Egger's test), confirming an absence of significant publication bias.

## Discussion

The main finding from this meta-analysis is the observation of a small, but statistically significant increase in the variability of GSH concentration in the ACC among patients with schizophrenia compared to healthy control subjects. This result was not attributable to publication bias, or a significant between-studies heterogeneity. Thus, we confirm the hypothesis that ACC GSH levels become notably variable among individuals with schizophrenia, adding credence to the existence of subgroups with both increased and decreased GSH within the diagnostic category of schizophrenia. We also note that there was no significant mean reduction in GSH levels when considering patients with schizophrenia as a single homogenous group. There was a low to medium between-studies heterogeneity (I^2^ <50%) in the mean effect estimate; but this was not driven by any single study, again adding to the argument that between subjects' variation may contribute to the dispersion of the mean effect in MRS studies of ACC GSH.

**Figure 2:**
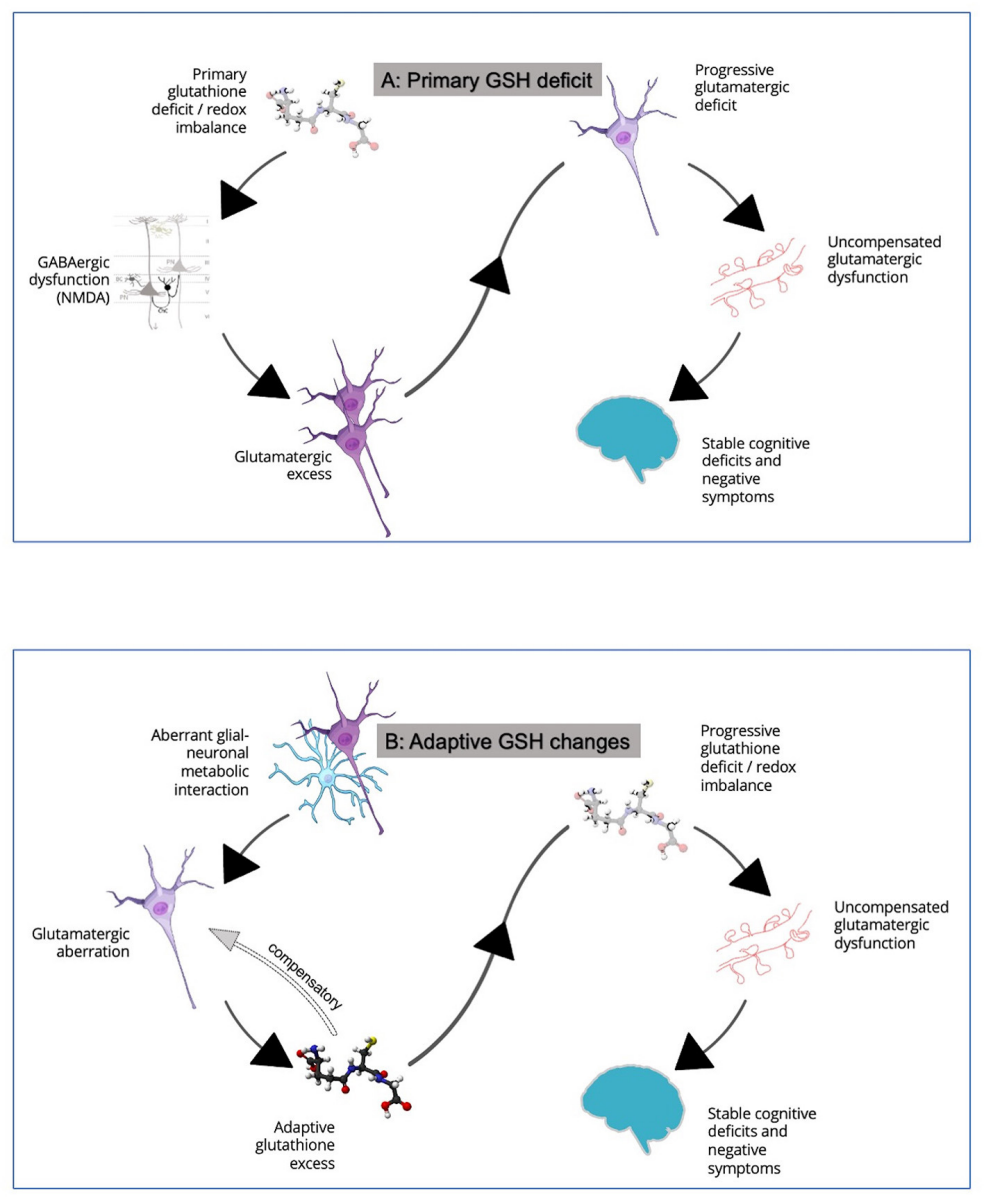
**(A)** In this model, a primary defect in glutathione generation is thought to contribute to NMDA hypofunction in cortical microcircuits, leading to relative glutamatergic excess via disinhibition of pyramidal neurons. This induces a glutamatergic excess state. As excitotoxic damage ensues, loss of dendritic spines and a longer-term picture of cognitive deficits and negative symptoms develop. See Steullet et al. (35) for more details. **(B)** In this model, GSH changes are considered to be adaptive consequences of glutamatergic aberrations. Primary glutamatergic deficit, likely originating from aberrant neuron-glia interactions, leads to an early GSH excess via diversion of GSH-glutamate metabolic pathways. A two-way interaction between GSH and glutamate may serve to counteract both glutamatergic deficit and excess in early stages. This adaptive response restores glutamate to near normal levels as a compensatory mechanism. But this cannot be sustained for long especially as neural activity diminishes and GSH reserves are depleted, with loss of dendritic spines and a longer-term picture of cognitive deficits and negative symptoms. See Koga et al. (36), and Sedlak et al. (37), for how GSH levels may relate to glutamate and Limongi et al. (38) for observations relating to opposing effects of anterior cingulate GSH and glutamate on effective connectivity. The relationship between GSH and glutamate is discussed in detail elsewhere (16). Credits for images used in this figure: Scidraw.io doi: 10.5281/zenodo.4421165; 10.5281/zenodo.3926048; 10.5281/zenodo.3926143; 10.5281/zenodo.3926604. Ball-and-stick model of the glutathione molecule from Ben Mills via Wikimedia commons.

Why should the GSH levels be more variable within the patient group? We see two possibilities here. Firstly, the increased among-individuals variability in GSH levels may result from the presence of a dynamic, time-varying course for GSH aberrations in schizophrenia. Such dynamic changes in putative biomarkers is a well-established aspect of schizophrenia (39). Given that GSH is a component of stress-response system, acute, untreated symptomatic psychosis may relate to higher levels of GSH than stable clinical states of schizophrenia. This putative GSH excess may relate to the hypothesized excitotoxic surge in glutamate (40) that affects the redox balance. GSH surge may occur as a compensatory process to limit the excitotoxicity. We observed that in untreated first episode patients, an elevated ACC GSH at the time of presentation occurred in those with higher levels of thought disorder, in conjunction with reduced integrity of the adjacent white matter tract (cingulum) (41). Interestingly, such untreated symptomatic patients with higher GSH respond much faster to the regular antipsychotic treatments over the next 6 months (42). We also noted that while glutamate excess related to the degree of disinhibition within the dorsal ACC node of the Salience Network, GSH excess had the opposing effect, supporting the hypothesized compensatory function (38). In line with this evidence, we observe higher than expected GSH levels at the prodromal, clinical high-risk state of psychosis, in association with better social and functional outcomes (43). Second, as the trajectory of schizophrenia unfolds, the putative early compensatory GSH surge may not be sustained; instead a relative GSH-deficit state may ensue especially in patients who experience poorer outcomes such as higher residual symptom burden (34). When both high and low GSH phases are captured in a sample with some patients in an acute stage of excitotoxic compensation and others in the later “spent” stage of decompensation, this may increase sample heterogeneity as reported here. If this is the case, then longitudinal follow-up of patients with initially high GSH levels, should reveal a dynamic lowering of GSH to generate a deficit state over time. See Figure 2 where this scenario is described as “Adaptive GSH Changes.”

The second possible explanation of the increased variance is the presence of distinct subgroups with stable but different levels of GSH as a “trait” feature of schizophrenia. Support for this explanation comes from the existing longitudinal studies of ACC GSH in schizophrenia which do not reveal a picture of dynamic change in GSH levels. Instead, existing longitudinal MRS (44, 45) as well as genetic studies (31) indicate a constitutional deficit of GSH, at least in some individuals with schizophrenia. The modest effect-size of the reported GSH deficit even in the chronic and partially treatment-resistant samples (7, 46), suggests that either (1) only a small number of patients belong to the putative GSH-deficit (or redox-deficient) subgroup. (2) some individuals from the putative GSH-excess subgroup (or redox sufficient) may still progress to chronic or resistant stages of schizophrenia. In the context of our current observation of increased variation in GSH levels in schizophrenia, the extant literature supports the model of ‘Primary GSH Deficit' (Figure 2) in some individuals with schizophrenia; this deficit may be pathoplastic and influence the treatment outcomes [e.g., treatment resistance (47)] among patients. Demonstrating bimodal distribution of GSH levels in large samples of patients in early illness stages, in conjunction with clinical and functional validation of the distributed values, will add substantial evidence to our claim regarding the existence of subgroups (16).

In addition to the above considerations, it is also likely that the use of antipsychotics and other medications as well as a high degree of variability in lifestyle factors and daily functioning (including diet) may make the patient group more heterogenous. A detailed exposition of methodological approaches required to conclusively establish the presence of the deficient/sufficient subtype can be found elsewhere (16).

The lack of significant mean difference, despite a trend toward GSH-deficit in schizophrenia, compared to the two prior meta-analyses (7, 12) relates to several recent studies that failed to demonstrate a group difference for patients vs. healthy controls comparisons, and one study showing a relatively higher GSH levels in patients (6). This latter study (6) from our group, specifically recruited early stage, untreated patients from a first-episode service. Such patients typically have much better outcome compared to patients with much more established illness and long-term antipsychotic exposure. Irrespective of the mean change, an increase in GSH variability in schizophrenia reflects reduced within-group predictability of the adaptive antioxidant response when patients face increased oxidative stress. It is possible that patients adopt different stress-response strategies other than GSH-centered mechanisms when facing redox stress, generating variance in GSH levels. Quantifying the covariance between GSH and other critical intracortical markers of antioxidant pathways will be required in the future to investigate this notion.

We highlight several limitations of this meta-analysis of variance. We restricted our search to one brain region (ACC) as many studies in schizophrenia have focussed on this region; the variability may be higher or lower in other regions of the brain. Between studies, the anatomical definition of ACC voxel was variable, as discussed in our prior work (7). We also included only studies that focussed on patients with schizophrenia after the diagnosis; prodromal or high-risk states are characterized by notable morphological changes (48) but these were not captured in our study. Finally, higher variance in an illness-afflicted group may occur due to higher variability in the measurement approaches (e.g., higher variability in the MRS voxel placement in the patient group; head movement being higher among patients), which cannot be inferred directly from a meta-analytical synthesis. Head-to-head comparisons with other patient groups will be needed to infer specificity of this finding to schizophrenia.

Individual variance in oxidative stress response is a feature of schizophrenia and likely crucial in identifying the potential prognostic trajectory for individuals with this condition. Our findings raise two key questions. First is whether the MRS based subtyping of patients could assist interpreting outcomes of future antioxidant trials in this illness. Targeting GSH-deficient phenotypes with specific antioxidant interventions and studying downstream biological effects could improve our understanding of prognosis-determining factors that have a pathoplastic effect on schizophrenia. Second is the question of how oxidative stress based preclinical models of schizophrenia can be generated to capture the full spectrum of this illness. A representative model of schizophrenia that is true to its heterogenous clinical phenotype likely includes both redox-deficient and redox-sufficient individuals. We, like many other observers in the field (49), consider antioxidant system to be an important non-dopaminergic target for early intervention in schizophrenia; addressing the questions raised here will be of critical importance to realize the potential promise of this pathway in treating psychotic disorders.

## Author Contributions

LP conceived, directed the analysis, and wrote the first draft. PS undertook literature search and independently verified extracted data. XL undertook the statistical analysis under QL's supervision. LP, PS, XL, and QL contributed to the writing of the manuscript. All authors contributed to the article and approved the submitted version.

## Funding

This work was funded by the Canadian Institute of Health Research (Foundation Grant 375104 to LP), Bucke Family Fund (LP and PS) and the Academic Medical Organization of South Western Ontario (LP), the National Natural Science Foundation of China (No. 81873909 to QL), the Science and Technology Commission of Shanghai Municipality (No. 20ZR1404900 to QL), the Shanghai Municipal Science and Technology Major Project (Nos: 2018SHZDZX01 and 2021SHZDZX0103 to QL).

## Conflict of Interest

LP reports personal fees from Otsuka Canada, SPMM Course Limited, UK, Canadian Psychiatric Association; book royalties from Oxford University Press; investigator-initiated educational grants from Janssen Canada, Otsuka Canada outside the submitted work. PS reports speaker fee from Lundbeck Canada. The remaining authors declare that the research was conducted in the absence of any commercial or financial relationships that could be construed as a potential conflict of interest.

## Publisher's Note

All claims expressed in this article are solely those of the authors and do not necessarily represent those of their affiliated organizations, or those of the publisher, the editors and the reviewers. Any product that may be evaluated in this article, or claim that may be made by its manufacturer, is not guaranteed or endorsed by the publisher.
